# Tuning the intentional corona of cerium oxide nanoparticles to promote angiogenesis via fibroblast growth factor 2 signalling

**DOI:** 10.1093/rb/rbac081

**Published:** 2022-10-20

**Authors:** Lu Fu, Rupeng Li, John M Whitelock, Megan S Lord

**Affiliations:** Graduate School of Biomedical Engineering, UNSW Sydney, Sydney, NSW 2052, Australia; School of Minerals and Energy Resources Engineering, UNSW Sydney, Sydney, NSW 2052, Australia; Graduate School of Biomedical Engineering, UNSW Sydney, Sydney, NSW 2052, Australia; Graduate School of Biomedical Engineering, UNSW Sydney, Sydney, NSW 2052, Australia

**Keywords:** cerium oxide, nanoparticle, endothelial, angiogenesis

## Abstract

Inadequate angiogenesis is a hallmark of conditions including cardiovascular diseases, stroke and chronic diabetic wounds, which exhibit tissue ischaemia ensuring that therapeutic strategies to promote angiogenesis are of great interest. However, many angiogenic treatments involve the delivery of growth factors which have limited clinical success due to poor stability, high manufacturing cost and poor efficacy. Cerium oxide nanoparticles (nanoceria) can either promote or inhibit angiogenesis depending on their surface corona chemistry. Here, nanoceria were functionalized with an intentional heparin corona, a polysaccharide which binds and signals growth factors, of different chain lengths and surface grafting density to establish their effect on angiogenesis. These nanoparticles promoted angiogenesis *in vivo* with the surface grafting density positively correlated with angiogenesis over the widest concentration range; however, chain length did not play a role. The heparin–nanoceria supported fibroblast growth factor 2 (FGF2) signalling *in vitro* and promoted FGF2-mediated angiogenesis *in vivo*. The nanoparticles were internalized by endothelial cells *in vitro* where they trafficked to the lysosomes and reduced cell viability suggesting that the angiogenic activity of heparin–nanoceria is mediated in the extracellular environment. Together, this study adds to our knowledge of the angiogenic effects of heparin–nanoceria towards finding new angiogenic treatments.

## Graphical Abstract

**Figure:**
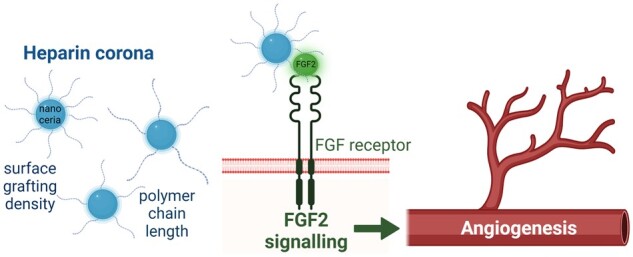
Graphical abstract created with BioRender.com.

## Introduction

Blood vessels are located throughout the body allowing the exchange of oxygen, nutrients, carbon dioxide and waste. Angiogenesis, or neovascularization, is the formation of new blood vessels from the pre-existing vasculature, a process that is finely controlled by the balance between endogenous promotors and inhibitors [1]. Insufficient angiogenesis is a hallmark of cardiovascular diseases, stroke and chronic diabetic wounds which exhibit tissue ischaemia and hypoxia [2, 3]. Thus, therapeutic strategies to promote angiogenesis are of great interest.

Angiogenesis is supported by various factors, the most potent of which are vascular endothelial growth factor (VEGF) and fibroblast growth factor 2 (FGF2) [4, 5] that are up-regulated in response to hypoxia and regulate hypoxia-inducible factor 1-alpha [6]. These growth factors bind to their activated cognate receptors on the endothelial cell surface, VEGF receptor 2 (VEGFR2) and FGF receptor 1 (FGFR1), respectively, which stimulate the proliferation, differentiation, migration and survival of endothelial cells [7, 8].

Exogenous growth factor delivery has been explored as a therapeutic angiogenic strategy; however, performance is sub-optimal due to poor stability, high cost related to the low manufacturing yield and poor efficacy [9]. Recently, protein engineering approaches have been applied to growth factors to enhance the binding and signalling duration at the cell surface; however, these approaches do not overcome the high manufacturing costs [10–12]. Similarly, cell-based approaches are costly and with low efficacy [13, 14]. Thus, new approaches to stimulate angiogenesis are needed.

Cerium oxide is a rare earth metal that exhibits redox properties which mimic the activities of catalase (CAT) and superoxide dismutase (SOD), making it an attractive reactive oxygen species (ROS) modulating nanomaterial for biomedical application [15–18]. Hypoxia increases intracellular ROS and while moderately elevated ROS levels activate redox-sensitive signalling pathways to induce angiogenesis, high levels of ROS are harmful for tissues via the induction of oxidative stress [19, 20]. Cerium oxide nanoparticles (nanoceria) have been shown to either stimulate or inhibit angiogenesis depending on their surface corona chemistry [21–24]. Thus, there is a need to further tune the surface properties of nanoceria for their desired application.

Surface functionalization is recognized to improve the biological performance of nanoparticles [18]. A variety of ligands have been intentionally immobilized on the surface of nanoceria including PEG, citrate and heparin; coronas which increase colloidal stability and modulate biological properties [25–28]. However, we are yet to establish the relationship between corona chain length and surface grafting density and changes in the biological properties of nanoceria [18].

Heparin is a clinically used polysaccharide widely explored for drug delivery applications due to its ability to bind many growth factors, including FGF2 and selected isoforms of VEGF, to potentiate their activity [29]. These activities of heparin mimic heparan sulphate in most tissues [30–32]. For example, the combined delivery of VEGF and FGF2 in heparin functionalized biomaterials is reported to promote angiogenesis *in vivo* [33]. Thus, heparin functionalization is a promising approach to confer biomaterials with angiogenic activity.

Here, we synthesized nanoceria and introduced intentional heparin surface coronas of different polymer lengths and surface grafting density. We employed *in vivo* and *in vitro* assays to establish the effect of corona grafting density and polymer length on the angiogenic properties of nanoceria. Such knowledge is expected to enable progress in the biomedical applications of nanoceria including as new treatments for ischemic diseases.

## Experimental section

### Reagents and materials

All reagents were purchased from Sigma-Aldrich (Castle Hill, Australia) unless stated otherwise.

### Synthesis of cerium oxide nanoparticles (nanoceria)

Nanoceria were synthesized via precipitation using high purity cerium (III) nitrate hexahydrate (99.999%) and sodium hydroxide as starting materials as described previously [34–36]. Briefly, sodium hydroxide (0.05 M) was added dropwise to cerium (III) nitrate hexahydrate (0.01 M) in stoichiometric excess (1:5 ratio of CeNO_3_.6H_2_O: NaOH) via a peristaltic pump (2 ml/min) to the stirred solution. The precipitate was centrifuged and washed five times with Milli-Q water with the supernatant removed after each wash until the pH of the supernatant reached 7.0. The resulting precipitate was dried at 100°C for 24 h.

### Heparin functionalization of nanoceria

Nanoceria were functionalized with 3-aminopropyltriethoxysilane (APTES) as described previously [35]. Briefly, nanoceria (10 mg/ml) were dispersed in anhydrous dimethyl formamide (DMF) and APTES (0.25 ml, 1.07 × 10^3^ mol) was added dropwise to the nanoceria suspension. The flask was resealed with a rubber septum and stirred overnight at 45°C. The APTES-modified particles were washed four times with DMF, and acetone was used for the final wash to facilitate the removal of DMF. The particles were dried in a vacuum desiccator at room temperature for 2 days.

Heparin was conjugated to APTES–nanoceria as described previously [35]. Briefly, unfractionated heparin from porcine sub-intestinal mucosa (Hep, average molecular mass 18 000 Da) or low-molecular-weight heparin (L-hep, average molecular mass 6276 Da, BOC Sciences, NY) was activated by first dissolving into a 1:1 dimethyl sulphoxide (DMSO)/water mixture (10 ml). EDC and NHS were then added and stirred at 50°C for 6 h. The heparin–NHS was added to the suspension containing APTES–nanoceria in 1:1 DMSO/water mixture and stirred for 3 days at room temperature. Different amounts of Hep (33 mg; Hep–nanoceria) and L-hep (11 mg for L-hep–nanoceria-A and 33 mg for L-hep–nanoceria-B) were used with the same amount of APTES–nanoceria (150 mg) to produce different heparin surface grafting densities. The resulting heparin functionalized nanoceria were washed twice with 1:1 DMSO/water mixture, followed by five washes with Milli-Q water. The particles were transferred to a vacuum desiccator for further drying at room temperature for 2 days.

Alexafluor 488-conjugated nanoceria preparations were prepared by mixing APTES–nanoceria or heparin functionalized nanoceria (20 mg) with Alexafluor 488 NHS Ester (0.17 mg; ThermoFisher Scientific, Australia) in DMSO, followed by incubation at room temperature for 4 h with continuous stirring. The particles were washed twice with acetone and three times with Milli-Q water, and then were dried in a vacuum desiccator at room temperature for 2 days.

### Characterization of nanoparticles

#### Transmission electron microscopy

Nanoparticle size and morphology were determined by high-resolution transmission electron microscopy (HR-TEM; CM200, Philips, The Netherlands) performed at 200 kV with a SIS CCD camera. Samples were dispersed in ethanol (80 w/v %) and sonicated for 10 min at room temperature and then transferred to a porous carbon film supported on a copper grid and air dried prior to imaging.

#### X-ray diffraction

X-ray diffraction (XRD; X’Pert MPD, Phillips, The Netherlands) analysis was performed to determine crystallite characteristics using a Cu-Kα radiation source (λ = 1.54060 Å) and a pixel array detector which scanned in the 2θ range between 20 and 80° with a 0.026° step size. Rietveld refinement of the XRD profiles was performed with the PANalytical X’pert HigScore Plus software and crystallite characteristics were determined with reference to cerianite. The average crystallite size (*d_XRD_*) was calculated using Scherrer’s formula: $d_{\mathrm{XRD}}=K\lambda /\beta \;\text{cos}\;\;\theta$ [37], where K = Scherrer constant = 0.943, λ = wavelength of the X-ray (nm), β = full width at half maximum (FWHM) and θ = diffraction angle of the primary peak (1 1 1) (nm).

#### X-ray photoelectron spectroscopy

Elemental composition was determined by X-ray photoelectron spectroscopy (XPS; ESCALAB250Xi, ThermoFisher Scientific). A monochromated Al K alpha x-radiation (1486.68 eV) at 120 W was used. The high-resolution narrow spectra were recorded with electron pass energy of 20 eV for region scans, to achieve the maximum spectral resolution. The binding energy of the Au 4f7/2 at 83.96 eV, Ag 3d5 at 368.21 eV and Cu2p3 at 932.62 eV were used to calibrate the binding energy scale of the spectrometer. XPS spectra of Ce3d were analysed for the area under each peak and the Ce^3+^/Ce^4+^ ratio was calculated [38]. Further structural detail was determined by Raman spectroscopy (inVia, Renishaw, UK), which was performed with a 25 mW He-Cd laser with an excitation wavelength of 532 nm. Spectra were recorded using the extended measurement mode in the range 300–700 cm^−1^ with 10 accumulations and 1800 l/mm grating. The particle size was estimated from the FWHM of the nanoceria active peak [39].

#### Dynamic light scattering

Aggregate size was determined by dynamic light scattering (DLS; Star Zetasizer Nano ZS, Malvern, UK) performed on samples (1.5 μg/ml in PBS, pH 7.4) that were sonicated prior to analysis. Measurements were taken using a 633 nm laser at 25°C for 120 s with automatically set laser position and attenuation. Zeta potential (Zetasizer Ultra, Malvern, UK) was measured (average of 60 runs per analysis) for samples (50 μg/ml in Milli-Q water, pH 7) that were sonicated prior to analysis.

#### Attenuated total reflectance Fourier-transform infrared spectroscopy

Surface chemistry was assessed by attenuated total reflectance Fourier-transform infrared spectroscopy (ATR-FTIR; Spotlight 400, PerkinElmer, MA, USA) measured at 4 cm^−1^ resolution with 50 scans over the range of 600–4000 cm^−1^. Thermal gravimetric analysis (Q500, TA instruments, DE, USA) was performed in a nitrogen atmosphere (purged/balanced at 25/15 ml/min) with samples placed on a platinum pan. The samples were heated to 1000°C at a rate of 10°C/min with the change in mass recorded and used to calculate the extent of APTES and heparin functionalization [40].

### SOD mimetic activity

The SOD assay kit was used as per the manufacturer’s instructions. Briefly, nanoparticle suspensions (20 μl; final concentration of 1.5–1000 μg/ml in Milli-Q water) were added to wells of a 96-well plate and mixed with 2-(4-iodophenyl)-3-(4-nitrophenyl)-5-(2,4-disulfophenyl)-2H-tetrazolium, monosodium salt (WST-1; 200 μl). The reaction was initiated with the addition of xanthine oxidase (20 μl; enzyme (15 μl) in the dilution buffer (2.5 ml)). After incubation for 20 min at 37°C, the absorbance at 450 nm was measured using a plate reader (Synergy HTX multi-mode reader).

### CAT mimetic activity

The CAT assay kit (ThermoFisher, catalogue number A22180) was used as per the manufacturer’s instructions. Briefly, nanoparticle suspensions (20 μl; final concentration of 1.5–1000 μg/ml in Milli-Q water) were added to wells of a 96-well plate followed by the addition of 40 μM H_2_O_2_ (25 μl) to each well. After incubation for 20 min at room temperature, the Amplex Red reagent (10-acetyl-3,7-dihydroxyphenoxazine)/horseradish peroxide working solution (50 μl) was added in each well and then the plate was incubated for 30 min at 37°C. The fluorescence was measured at excitation/emission wavelengths of 540/600 nm using a plate reader.

### *In vivo* analysis

#### Chicken chorioallantoic membrane angiogenesis assay

Chicken chorioallantoic membrane (CAM) protocols were approved by the UNSW Animal Care and Ethics Committee (ACEC 18/17A and 18/16A) and performed as described previously [41]. Control conditions included baseline (PBS or 25 ng/ml FGF2) and positive (100 ng/ml FGF2 or VEGF165) controls. Samples included nanoceria alone (100–1000 μg/ml; equivalent to 5–50 μg/egg) or together with FGF2 (25 ng/ml). Images were analysed for blood vessel density using software developed in-house to perform blood vessel segmentation [42], convert the grey-scale image to a binary image by thresholding to minimize the intraclass variance of the thresholded black (background) or white (blood vessels) pixels. The noise in the binary image was reduced by removing connected components that had fewer than 50 pixels using the morphological operation known as area opening. Finally, the blood vessel density was calculated by the number of white pixels divided by the total number of pixels in the image.

For histological analysis, samples were fixed in 4% paraformaldehyde (PFA) for 72 h at 4°C and embedded in paraffin before being sectioned (5 μm thick). Sections were deparaffinized using xylene (twice, 5 min each) and the graded ethanol washes (twice in 100% (v/v), once in 95% (v/v), once in 70% (v/v), 3 min each) followed by a deionized water rinse for 3 min. The slides were stained with haematoxylin (Vector Laboratories) for 5 min and then rinsed in deionized water until the water was colourless. The slides were then incubated in acid alcohol for 10 s followed by 10 s in tap water. The slides were incubated in bluing solution for 2 min followed by rinsing with deionized water and then stained with eosin (Fronine) for 2 min. The slides were dehydrated by graded ethanol washes (once in 90% (v/v), twice in 100% (v/v), 1 min each) followed by two washes with xylene for 5 min each followed by mounting and digital imaging (Aperio ScanScope XT scanner).

### *In vitro* analysis

#### Human endothelial cell culture

Human umbilical vein endothelial cells (HUVEC; Lonza, Australia) were cultured in endothelial cell growth medium with 2 (v/v) % foetal bovine serum (EGM-2; Lonza, Australia) at 37°C with 5% CO_2_.

### Cytotoxicity

HUVECs (10^4^ cells/well) were seeded into wells of a 96-well cell culture plate and incubated for 16 h prior to the addition of nanoceria (0–200 μg/ml) in EGM-2 media. After 24 or 72 h, wells were washed three times with PBS and processed for the CyQuant assay (ThermoFisher Scientific) as per the manufacturer’s instructions. Background measurements were obtained for each treatment in the absence of cells.

### FGF2 signalling

BaF32 cells stably transfected with fibroblast growth factor receptor 1c are a model system to identify active ternary complexes between the receptor, FGF2 and glycosaminoglycans, which is measured by cell proliferation. The assay was set up as described previously [43] with controls (medium or 30 nM Hep or L-hep) and samples equivalent to 30 nM heparin (19.852 μg/ml Hep–nanoceria, 25 μg/ml L-hep–nanoceria-A, 5.23 μg/ml L-hep–nanoceria-B). The number of viable cells was analysed by flow cytometry. Samples were mixed with calibration particles (1 μl; SPHERO^TM^ drop delay calibration particles (Spherotech Inc., Lake Forest, IL) together with PI (2 μg) and incubated for 5 min before analysis in the flow cytometer (for 10^4^ gated cell and bead events. Data were analysed using the FlowJo_V10 software with gates for live and dead cells applied based on PI. Total cell number was determined by:

$$\mathrm{Cell}\;\mathrm{concentration}\;=\frac{\mathrm{number}\;\mathrm{of}\;\mathrm{cell}\;\mathrm{events}}{\mathrm{number}\;\mathrm{of}\;\mathrm{bead}\;\mathrm{events}}\times \mathrm{bead}\;\mathrm{concentration}$$

### Visual assessment of the interactions between nanoparticles and BaF32 cells

Coverslips (ThermoFisher Scientific, Australia) were cleaned with ethanol (80% v/v) for 16 h and then dried followed by coating with poly-D-lysine (1 mg/ml) for 1 h at room temperature. The coverslips were then washed thrice with sterile Milli-Q and then dried for 2 h before BaF32 cells (10^4^ cells/well in 400 μl of medium) were seeded in eight-well silicone chambers placed on the poly-D-lysine coated coverslips. Cells were incubated for 16 h at 37°C before being treated with nanoparticles (1.5 µg/ml) dispersed in medium for 24 h. Cells were then stained with CellMask™ Orange Plasma membrane Stain (1:500; ThermoFisher Scientific) and Hoechst 33342 (1:2000 in PBS; ThermoFisher Scientific, Australia) and incubated for 10 min at 37°C followed by two washes with PBS. Cells were then fixed with formaldehyde (4% w/v) in PBS for 15 min at room temperature and then washed twice with PBS. Cells were then imaged by confocal microscopy (SP8, Leica, Wetzlar, Germany).

### Intracellular ROS and nanoparticle association with cells

Intracellular ROS was measured using 2′, 7′-dichlorodihydrofluorescein diacetate (DCFH-DA), a membrane-permeable dye that becomes fluorescent when oxidized by H_2_O_2_ and widely used to study the ROS scavenging capability of nanoceria [44, 45]. Cells (10^5^ cells/well in 1 ml of medium) were seeded into 12-well plates and incubated for 16 h at 37°C before the addition of the nanoparticles (1.5 μg/ml; 15 pg/cell) or equivalent amounts of heparin (0.041 μg/ml Hep, 0.011 μg/ml L-hep-A or 0.054 μg/ml L-hep-B). Cells were harvested after 24 or 72 h using TrypLE™ Express Enzyme (1×) (ThermoFisher Scientific). Cells were resuspended in flow buffer (2 mM EDTA, 0.5 w/v% bovine serum albumin (BSA) in PBS) and 10^5^ cells were resuspended in DCFH-DA (0.5 μM in flow buffer) and incubated for 20 min at 37°C before being washed with and resuspended in flow buffer (200 μl). Propidium iodide (PI; 2 μg) was added to each tube and mixed for 5 min before samples were analysed in a flow cytometer (LSRFortessa™ SORP, BD, Franklin Lakes, NJ) for fluorescence intensity (DCF and PI), forward scatter and side scatter (SSC) for 10^4^ cells after gating for live cells. Data were analysed with the FlowJo_V10 software (BD).

### Nanoparticle uptake analysis

Cells (10^4^ cells/well in 400 μl of medium) were seeded into eight-well chamber 1.5 borosilicate coverslips (ibidi GmbH, Germany) and incubated for 16 h at 37°C before being treated with nanoparticles (1.5 µg/ml) dispersed in medium for 24 h. The cells were then washed twice with PBS and stained with CellMask™ Deep Red Plasma membrane stain (1:500 diluted in medium; ThermoFisher Scientific) for 5 min at 37°C in the dark. The cells were then washed twice with PBS and the stained with LysoTracker^®^DND-99 (75 nM diluted in medium; ThermoFisher Scientific, Australia) for 1 h at 37°C in the dark. The cells were washed twice with PBS and fixed in formaldehyde (4% w/v) in PBS for 15 min at room temperature in the dark. The cells were then washed twice in PBS. The colocalization of Alexa Fluor 488-nanoceria with lysosomes was investigated using confocal microscopy (LSM 780, objective NA =1.4, Zeiss, Germany) and ZEN 2012 software (Zeiss). Fiji (ImageJ) was used to analyse the images with the plugin ‘Just Another Colocalization Plugin’. Thresholds were manually set, and the Thresholded Mander’s Colocalization Coefficient was obtained, where M1 is defined as the ratio of the ‘summed intensities of pixels from channel 1 for which the intensity in channel 2 is above zero’ to the ‘total intensity in channel 1’, and vice versa for M2 [46].

Cells were grown in 16-well chamber slides (ThermoFisher) for 4 h before the addition of medium containing nanoparticles (1.5 μg/ml). The cells were incubated for 24 h, washed twice with PBS and then fixed with 4% PFA in PBS for 15 min at 37°C. The cells were then washed twice with PBS to remove remaining PFA, permeabilized with 300 mM sucrose, 50 mM NaCl, 3 mM MgCl_2_, 2 mM HEPES and 0.5% Triton X-100, pH 7.2, in Milli-Q water for 5 min at 4°C and then incubated with BSA (1 w/v%) for 16 h at room temperature. Wells were then washed twice with 0.1 w/v % Tween in PBS (PBST) before incubation with Rhodamine-phalloidin (1:200 in PBST; ThermoFisher) for 1 h at room temperature in the dark. After three rinses with PBST, the cells were visualized by confocal microscopy (Leica SP2, 63× oil immersion objectives).

### VEGFR2 expression

Cells were prepared as in Section ‘Intracellular ROS and nanoparticle association with cells’ and incubated with either mouse anti-human VEGFR2 antibody (3.5 µg/ml in flow buffer; BioLegend, CA, USA) or isotype control (3.5 µg/ml in flow buffer; BD) for 30 min at 4°C. Samples were then washed twice with flow buffer and incubated with AlexaFluor488 conjugated secondary antibody (4 μg/ml in flow buffer; ThermoFisher Scientific) for 30 min at 4°C. Then samples were washed twice with and resuspended in flow buffer (200 μl) before being analysed in the flow cytometer.

### Statistical analysis

All results were presented as mean ± standard deviation (SD). One- or two-way analysis of variance followed by Tukey’s or Šídák’s Honestly Significant Differences was performed to compare statistical differences between different conditions after establishing that the data were normally distributed. A *P *<* *0.05 was considered significant. Analysis was performed using GraphPad Prism version 9 (GraphPad Software, San Diego, CA).

## Results and discussion

### Characterization of heparin functionalized nanoceria

**Figure 1.**
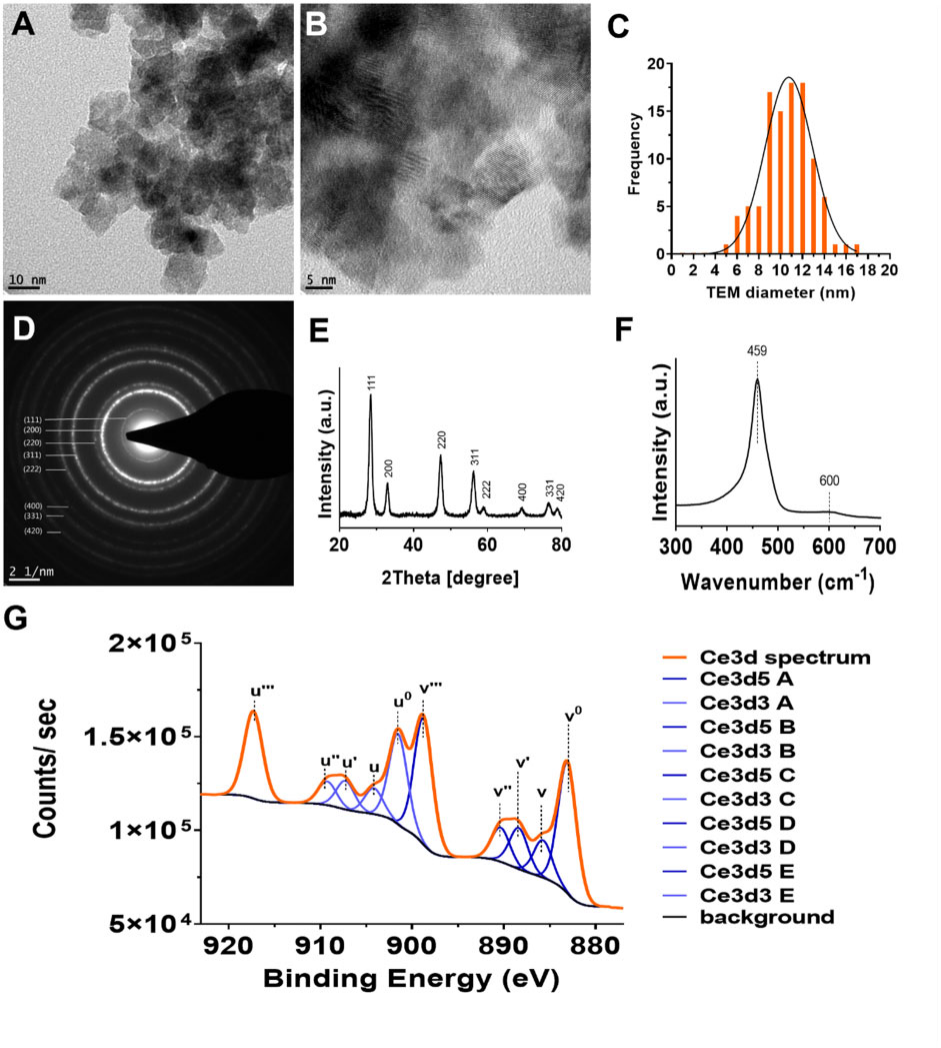
Characterization of nanoceria. (**A** and **B**) HR-TEM images. (**C**) Frequency histogram of nanoparticle diameter measured from TEM images. (**D**) Selected area electron diffraction (SAED) pattern. Scale bar = 2 nm^−1^. (**E**) XRD profile with peak assignment for cerium oxide. (**F**) Raman spectrum. (**G**) XPS spectrum of Ce3d of nanoceria with Ce^3+^ and Ce^4+^ peaks (denoted u, u′′, u′′′, v, v′′, v′′′ and u^0^, u′, v^0^, v′, which correspond to Ce^3+^and Ce^4+^, respectively).

Although the nanoceria aggregated under the dry conditions required for HR-TEM, their faceted morphology and well-defined lattice fringes was observed (Fig. 1A and B), which enabled estimation of the average crystallite size of 11.1 ± 2.2 nm (Fig. 1B and C and Table 1). In addition, the nanoceria exhibited high crystallinity with well-pronounced Debye–Scherrer diffraction rings in the selected area electron diffraction pattern assigned to the reflections (1 1 1), (2 0 0), (2 2 0), (3 1 1), (2 2 2), (4 0 0), (3 3 1) and (4 2 0) indicative of face-centred cubic nanoceria (Fig. 1D). XRD indicated the presence of crystalline cerium oxide with a 96% compositional match with the cerium oxide standard (JCPDS data card no: 00-043-1002). In addition, the primary particle size was estimated as 11 nm from XRD (Fig. 1E and Table 1). Raman spectroscopy also indicated the purity of nanoceria with a single major peak at 459 cm^−1^ attributed to the symmetric O–Ce–O stretching and the weak peak around 600 cm^−1^ attributed to oxygen defects in the cerium lattice [47] (Fig. 1F), while the primary particle size estimated by this technique was 5.6 nm (Table 1) in line with a previous report [36]. XPS indicated the presence of elements associated with nanoceria including cerium and oxygen.

**Table 1.** Properties of nanoceria and heparin functionalized nanoceria

		Nanoceria	Hep–nanoceria	L-hep–nanoceria-A	L-hep–nanoceria-B
Primary particle size (nm)	XRD	11	–	–	–
	TEM	11.1 ± 2.2	10.6 ± 2.7	11.8 ± 1.3	10.6 ± 1.3
	(*n* = 10–100)				
	Raman	5.6	–	–	–
Ce^3+^/Ce^4+^ ratio		0.54	0.55	0.58	0.59
Surface functionalization	Hep/L-Hep (μmol/g)	–	1.5	1.8	5.7
	Hep/L-Hep (mg/g)	–	27	7.4	36

The Ce3d spectrum indicated that the nanoceria contained 35% of the cerium ions in the Ce^3+^ state (peaks u^0^, u′, v^0^, v′) with the remainder in the Ce^4+^ state (peaks u, u′′, u′′′, v, v′′, v′′′) which resulted in a Ce^3+^/Ce^4+^ ratio of 0.54 (Fig. 1G; Table 1; Supplementary Table S1). Together, these data indicated that the nanoceria were synthesized with high purity and a narrow size distribution.

**Figure 2.**
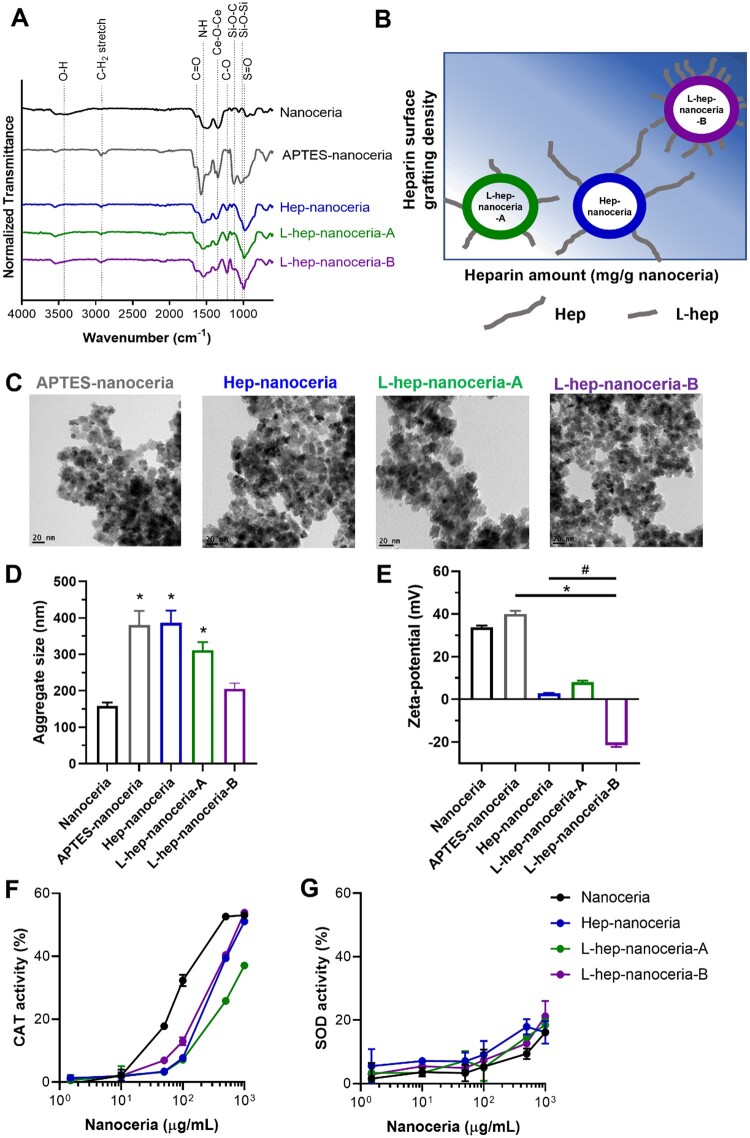
Characterization of heparin functionalized nanoceria. (**A**) ATR-FTIR spectra of nanoceria, APTES–nanoceria and heparin functionalized nanoceria. (**B**) Schematic representation of the relative amount and surface grafting density of heparin in hep–nanoceria, L-hep–nanoceria-A and L-hep–nanoceria-A. (**C**) Representative TEM images of APTES–nanoceria, hep–nanoceria, L-hep–nanoceria-A and L-hep–nanoceria-B. (**D**) Aggregate size of nanoceria, APTES–nanoceria and heparin functionalized nanoceria determined by DLS in PBS, pH 7.4. (**E**) Zeta potential of nanoceria, APTES–nanoceria and heparin functionalized nanoceria in Milli-Q water. (**F**) CAT mimetic activity. (**G**) SOD mimetic activity. Data are mean ± SD (*n* = 3). *P*‐values were calculated using one‐way ANOVA with Tukey’s test, *P <* *0.05 compared to nanoceria and ^#^*P < *0.05 compared to APTES–nanoceria.

Functionalization of nanoceria with APTES yielded changes in the corona chemistry as observed by ATR-FTIR with increased intensity of transmittance lines associated with CH_2_, N–H, Si–O–Si and Si–O–C bonds (Fig. 2A). Nitrogen and silicon were detected in APTES–nanoceria via XPS, while these elements were absent from nanoceria indicating the successful functionalization of nanoceria with APTES (Supplementary Table S2). Subsequent functionalization of APTES–nanoceria with Hep (Hep–nanoceria) or L-hep (L-hep–nanoceria-A and L-hep–nanoceria-B) further changed the corona chemistry as shown by ATR-FTIR with the increased intensity of transmittance lines associated with C–O and S = O corresponding to bonds within heparin (Fig. 2A). Sulphur was only detected in each of the heparin–nanoceria preparations when analysed by XPS which confirmed the successful conjugation of heparin onto APTES–nanoceria in Hep–nanoceria, L-hep–nanoceria-A and L-hep–nanoceria-B (Supplementary Table S2).

The extent of surface functionalization was measured by TGA and although the traces appeared quite similar for APTES–nanoceria and L-hep–nanoceria-A (Supplementary Fig. S1), the functionalization with L-hep requires modification of APTES at primary amino and carboxyl groups in heparin to form covalent bonds which will reduce the observable differences at low levels of heparin functionalization. Analysis of the TGA data indicated that Hep–nanoceria contained ∼1.5 μmol Hep/g nanoceria with a similar corona grafting density as L-hep–nanoceria-A (1.8 μmol L-hep/g nanoceria) (Table 1). However, L-hep–nanoceria-A contained less heparin than Hep–nanoceria due to conjugation with shorter heparin chains. In comparison, L-hep–nanoceria-B exhibited a 3-fold higher corona grafting density than L-hep–nanoceria-A with the same heparin polymer length (Table 1). Together, these heparin functionalized nanoceria enabled investigation of the mechanisms of biological activity based on heparin polymer length (Hep–nanoceria vs L-hep–nanoceria-A) and corona density (L-hep–nanoceria-A vs L-hep–nanoceria-B) (Fig. 2B) later in this study.

APTES and heparin functionalized nanoceria exhibited the same faceted and aggregated morphology as nanoceria (Figs 1A and B and 2C) with no change in average crystallite size compared to nanoceria (Supplementary Fig. S2 and Table 1). The hydrodynamic diameter of the nanoceria aggregates was 158 ± 9 nm (Fig. 2D) in line with a previous report [48], while Hep–nanoceria and L-hep–nanoceria-A formed significantly larger aggregates (*P *<* *0.0001 and *P *=* *0.0001, respectively). In contrast, L-hep–nanoceria-B formed aggregates that were not significantly different (*P *>* *0.05) in size to nanoceria (Fig. 2D and Supplementary Fig. S2). Conjugation of nanoceria with APTES increased the zeta potential, whereas heparin functionalization significantly reduced the zeta potential compared to both nanoceria and APTES–nanoceria (*P *<* *0.0001) with the nanoparticles containing the highest amount of heparin, L-hep–nanoceria-B, exhibiting the lowest zeta potential (Fig. 2E and Supplementary Fig. S2). Interestingly, given surface charge provides a steric repulsive force between particles leading to less aggregation [49], nanoceria and L-hep–nanoceria-B with a higher surface charge magnitude exhibited comparatively smaller hydrodynamic diameters than the other particles in this study. Aggregation of nanoceria is reported due to their small crystalline monomers and very high surface area with weak electrostatic interaction potentials [35, 50–53]. The results presented here indicate that post-synthesis, surface functionalization did not substantially alter the aggregation of the nanoceria that occurred during synthesis.

The Ce^3+^/Ce^4+^ ratios for nanoceria and all heparin functionalized nanoceria were similar (Supplementary Table S1), in agreement with previous studies [54]. The CAT and SOD mimetic activity assays were used to evaluate accessibility of the cerium oxide crystal lattice structure following surface functionalization. For all nanoparticles, the CAT mimetic activity increased with increasing particle concentration; however, there was a significant reduction in CAT activity (*P *<* *0.05) with heparin surface functionalization (Fig. 2F). The CAT-like activity is reported to positively correlate with the surface concentration of Ce^4+^ [17, 26] indicating that these remained accessible on the nanoceria surface following heparin functionalization, albeit at a lower level than for nanoceria. There was also a concentration dependence of the SOD mimetic activity; however there was no significant (*P *>* *0.05) difference with the heparin surface corona (Fig. 2G). This finding agrees with the higher proportion of Ce^4+^ than Ce^3+^ (Table 1) as previously reported [26].

### Nanoceria promote angiogenesis

**Figure 3.**
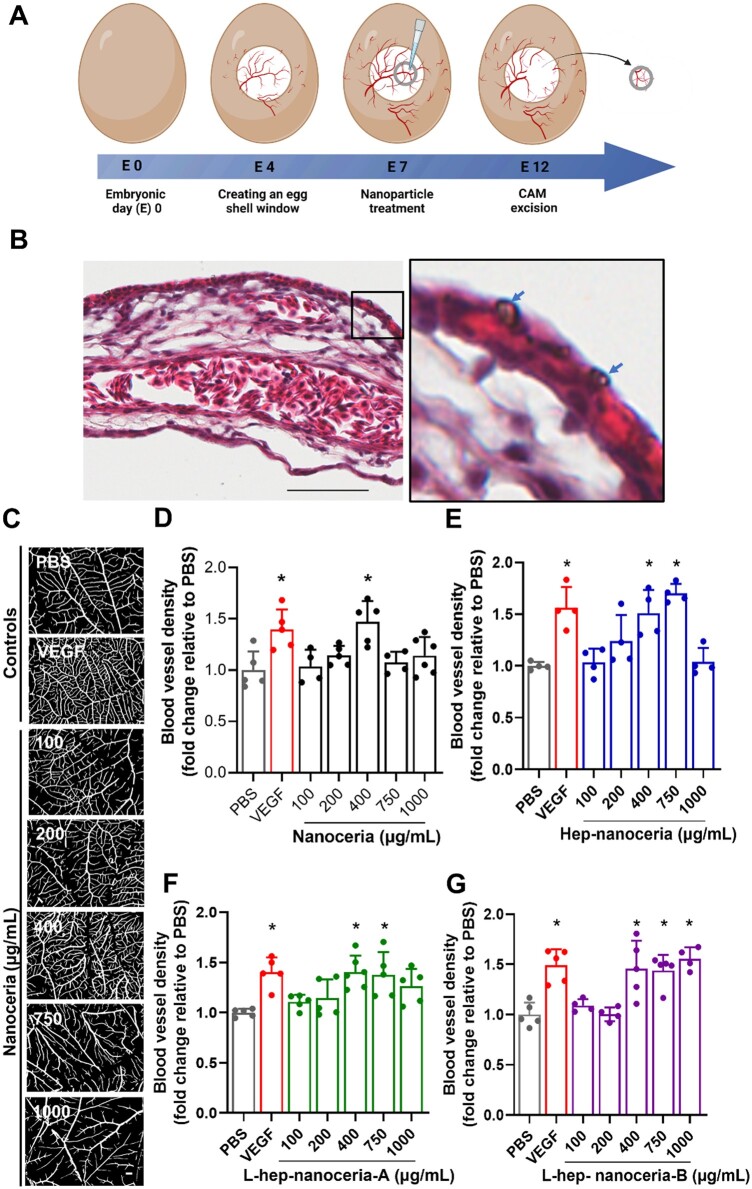
Nanoceria and heparin functionalized nanoceria promote angiogenesis *in vivo*. (**A**) Assay schematic. A window in the shell was created at embryonic Day 4 (E4), and samples were added to the CAM at E7 and the effect of the samples on the blood vessel density was analysed at E12. (**B**) Nanoceria localization to the surface membrane of the CAM analysed by H&E staining. Nanoceria are indicated by arrows. Scale bar = 50 μm. (**C**) Representative binary dissecting microscope images of the blood vessels in the CAM after exposure to PBS, VEGF165 (100 ng/ml, positive control) or nanoceria (100–1000 μg/ml). Scale bar = 500 μm. Blood vessel density relative to PBS for (**D**) nanoceria, (**E**) hep–nanoceria, (**F**) L-hep–nanoceria-A and (**G**) L-hep–nanoceria-B (100–1000 μg/ml). Data are mean ± SD (*n* = 4–6), *P*-values were calculated using one-way ANOVA with Tukey’s test. * *P *<* *0.05 compared to PBS.

The ability of the nanoceria to promote angiogenesis was explored in the CAM assay, a well-established model [55], where nanoparticles were added on embryonic Day 7 (E7) and the extent of blood vessel formation analysed on E12 (Fig. 3A). H&E staining at the end of the assay revealed that nanoceria aggregates were localized to the surface of the CAM (Fig. 3B); however, not all nanoparticles are likely to be resolved by this technique. Images of the CAMs exposed to nanoparticles (Fig. 3C) were analysed for blood vessel density (Fig. 3D–G) as a measure of angiogenesis. Nanoceria at a dose of 400 µg/ml resulted in a 1.5-fold increase in blood vessel density compared to PBS (*P *=* *0.0008), which was not significantly different to the positive control VEGF165 (*P *>* *0.05) (Fig. 3C and D). In contrast, none of the other nanoceria doses promoted angiogenesis compared to the control (Fig. 3C and D) indicating that nanoceria dose-dependently promoted angiogenesis in line with a previous report [50]. Like nanoceria, the heparin functionalized nanoceria promoted angiogenesis, but over a higher concentration range (Fig. 3D–G). Specifically, Hep–nanoceria at doses of 400 and 750 μg/ml promoted a 1.5- and 1.7-fold, respectively, increase in blood vessel density compared to PBS (*P *=* *0.0019 and *P *<* *0.0001, respectively) (Fig. 3E), while L-hep–nanoceria-A at the same doses increased blood vessel density by 1.4-fold each (*P *=* *0.0010 and *P *=* *0.0034, respectively) (Fig. 3F). Furthermore, L-hep–nanoceria-B at doses of 400, 750 and 1000 μg/ml increased blood vessel density 1.5, 1.4- and 1.6-fold, respectively (*P *=* *0.0007, *P *=* *0.0011 and *P *=* *0.0001, respectively) (Fig. 3G). These data indicated that high doses of nanoceria inhibited angiogenesis in this assay and that the heparin corona modulated this effect.

Endothelial cell proliferation is a key process in angiogenesis [56]. Thus, the ability of nanoceria and heparin functionalized nanoceria to support endothelial cell proliferation *in vitro* was analysed (Supplementary Fig. S3). At concentrations < 5 μg/ml, the nanoparticles supported endothelial cell growth to the same extent as the control over 24 h (Supplementary Fig. S3A). However, at concentrations > 5 μg/ml the nanoparticles reduced the endothelial cell number after 24 h compared to cells exposed to the control and with > 30% cell growth inhibition indicating cytotoxicity (*P *<* *0.05) (Supplementary Fig. S3A). However, while cytotoxicity has been reported for primary human endothelial cells [48, 57], it has not for nanoceria and heparin functionalized nanoceria (200 μg/ml) exposed to human monocytes [35] indicating differences between cell types. Thus, the nanoceria dose-dependently modulated endothelial cell proliferation and the heparin corona did not affect this activity. These results correlate with the dose-dependent angiogenic effect of the nanoceria in the CAM assay (Fig. 3).

Interestingly, when nanoceria were used in the CAM assay, they promoted angiogenesis at a dose of 400 µg/ml, while heparin functionalized nanoceria promoted angiogenesis over a wider dose range of 400–1000 µg/ml, which was likely mediated by potentiating endogenous growth factors via the heparin coating. Interestingly, L-hep–nanoceria-B, with both the highest amount and highest surface grafting density of heparin, promoted angiogenesis at doses of 400–1000 µg/ml. In contrast, Hep–nanoceria and L-hep–nanoceria-A with the same surface grafting density of heparin and overall less grafted heparin than L-hep–nanoceria-B promoted angiogenesis at doses of 400–750 µg/ml. These results indicate the fine balance of biological activities provided by the nanoceria and heparin components. At doses of 750 µg/ml and above, the promotion of angiogenesis was mediated by the heparin coatings but modulated by the nanoceria component, as nanoceria at this concentration did not promote angiogenesis. In the case of L-hep–nanoceria-B, the higher heparin surface grafting density likely masked the modulating effects of the nanoceria component to favour pro-angiogenic activity, while the pro-angiogenic activity of heparin in Hep–nanoceria and L-hep–nanoceria-A, which contained lower surface grafting density, were likely less effective in masking the modulating effects of the nanoceria component. Interestingly, the heparin doses within these nanoparticles were able to support angiogenesis at up to 50-fold lower doses than reported previously for heparin [58]. This suggests that the activity of heparin is enhanced when presented on the nanoceria, in line with previous analyses for other types of nanoparticles [59].

### Heparin functionalized nanoceria potentiate angiogenesis *in vivo* via FGF2

**Figure 4.**
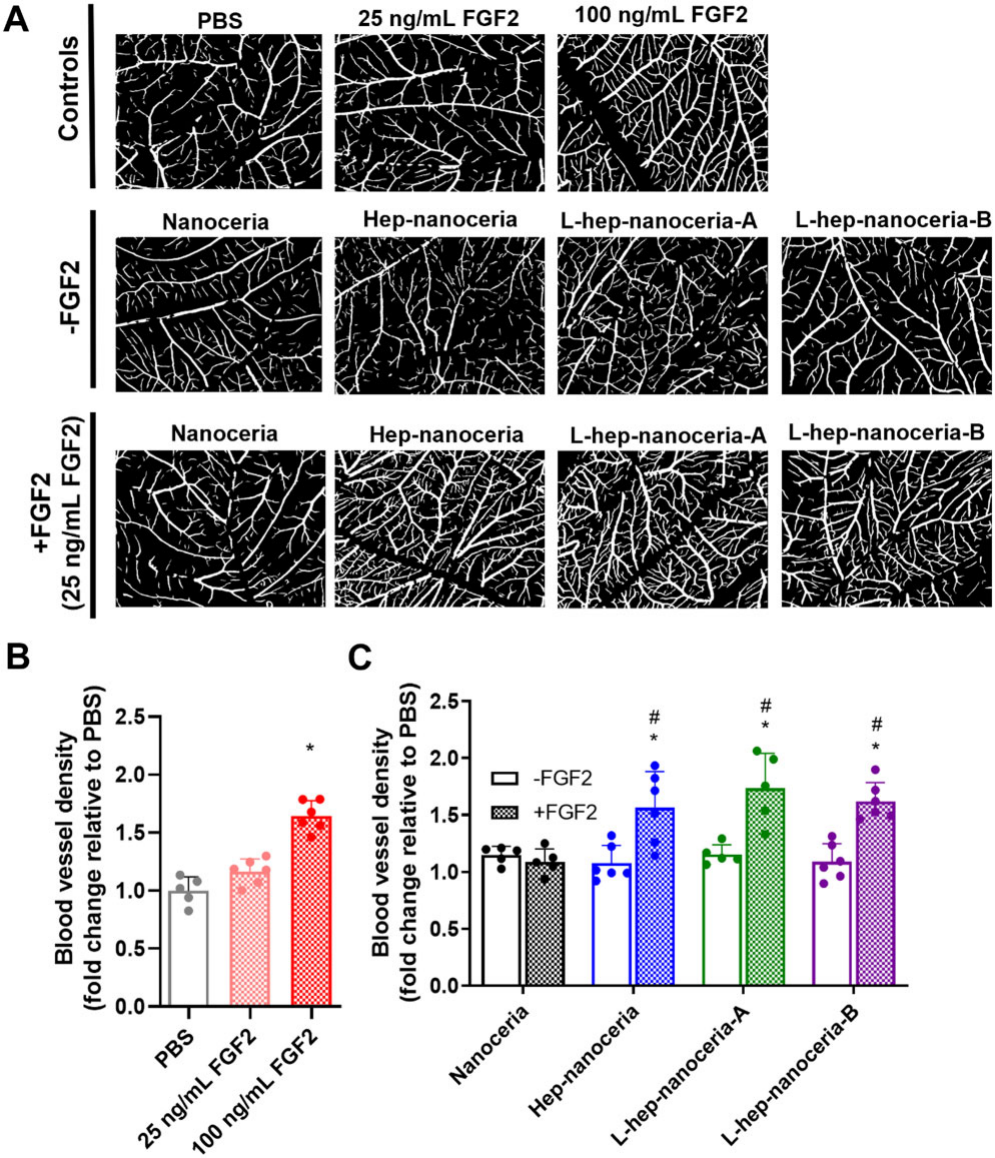
Heparin functionalized nanoceria potentiate FGF2 *in vivo*. (**A**) Representative binary dissecting microscope images of the CAMs exposed to controls (PBS, 25 ng/ml FGF2, 100 ng/ml FGF2) or test conditions (200 μg/ml nanoceria, hep–nanoceria, L-hep–nanoceria-A and L-hep–nanoceria-B) either alone or together with 25 ng/ml FGF2. Scale bar = 500 μm. (**B** and **C**) The images of the CAMs were analysed for blood vessel density. Data are expressed as fold change relative to PBS. Data are mean ± SD (*n* = 5–6), *P*-values were calculated using one-way ANOVA with Tukey’s test, *P <* *0.05 compared to PBS. ^#^*P *<* *0.05 compared to each treatment without FGF2.

Heparin binds and potentiates the signalling of angiogenic growth factors such as FGF2 [60] and this was explored in the CAM assay. The CAM was exposed to the nanoparticles (200 µg/ml) either in the presence or absence of FGF2 and images (Fig. 4A) were analysed for blood vessel density (Fig. 4B and C). FGF2 (100 ng/ml; positive control) increased blood vessel density 1.6-fold (*P *<* *0.0001) compared to PBS (negative control), whereas 25 ng/ml FGF2 did not support angiogenesis (*P *>* *0.05) (Fig. 4B). Nanoparticles alone (200 μg/ml) did not support angiogenesis (*P *>* *0.05); however, the combination of hep–nanoceria, L-hep–nanoceria-A or L-Hep–nanoceria-B with FGF2 (25 ng/ml) increased blood vessel density 1.6-, 1.7- and 1.6-fold, respectively, compared to CAMs exposed to PBS (*P *=* *0.0001, *P *<* *0.0001 and *P *<* *0.0001, respectively) (Fig. 4C). In contrast, there was no change in the blood vessel density for CAMs exposed to nanoceria in either the absence or presence of FGF2 (*P *>* *0.05) (Fig. 4C). These results indicated that the heparin corona on nanoceria potentiated FGF2 signalling *in vivo*. These findings are supported by reports that heparin binds FGF2 to induce angiogenesis [61]. Interestingly, there was no significant difference in the promotion of angiogenesis, as measured by blood vessel density, between the heparin–nanoceria preparations in the presence of FGF2 (Fig. 4C). These data indicated that neither the heparin corona surface density nor polymer length affected the level of angiogenesis mediated by exogenous FGF2 signalling *in vivo.*

**Figure 5.**
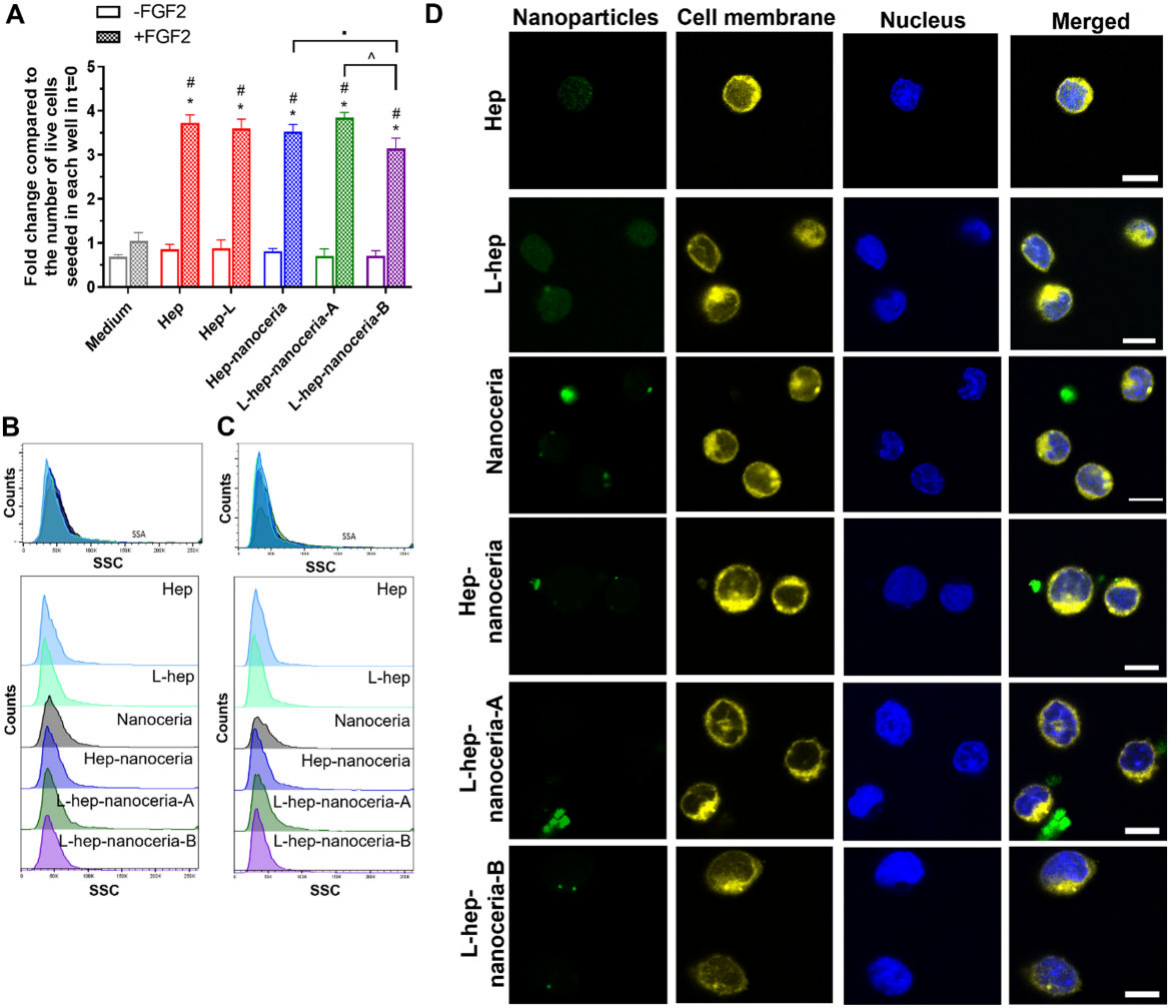
Heparin functionalized nanoceria bind and signal FGF2 at the cell surface. The formation of ternary complexes between heparin–nanoceria and FGF2 assayed using the BaF32 cell line expressing FGFR1c as measured by cell proliferation by flow cytometry. (**A**) Cells in the presence of FGF2 and heparin (either hep or L-hep; positive controls) were compared to cells in either the absence or presence of FGF and exposed to medium, hep–nanoceria, L-Hep–nanoceria-A or L-Hep–nanoceria-B after 72 h. Data were presented as fold change in live cell number compared to cells seeded in each well at 0 h (mean ± SD, *n* = 3), *P*-values were calculated using one-way ANOVA with Tukey’s test. *P < 0.05 compared to cells with FGF2. ^#^*P* < 0.05 compared to cells exposed to each of the treatments in the absence of FGF2. ^^^*P* < 0.05 between cells exposed to L-hep–nanoceria-A and cells exposed to L-hep–nanoceria-B. *P* < 0.05 of cells exposed to L-hep–nanoceria-B compared to cells exposed to hep–nanoceria. Flow cytometric analysis of nanoceria, heparin functionalized nanoceria and heparin interaction in the presence of FGF2 with BaF32 cells analysed by side scatter after (**B**) 24 or (**C**) 72 h of exposure. Data were displayed as the number of cells versus the SSC value. (**D**) Representative images of the interaction of nanoceria, hep–nanoceria, L-hep–nanoceria-A and L-hep–nanoceria-B (alexafluor 488 conjugated) in the presence of FGF2 with BaF32 cells (stained with CellMask for cytoplasm) and nucleus (stained with Hoechst 3393) after 24 h as measured by confocal microscopy. Scale bar = 10 μm.

Furthermore, the ability of the heparin functionalized nanoceria to potentiate the signalling of FGF2 was explored *in vitro* using BaF32 cells. BaF32 cells are derived from myeloid B cells that are IL-3-dependent, heparan sulphate proteoglycan deficient and transfected to only express FGFR1c on their cell membrane [62]. Thus, their proliferation occurs when either exposed to IL-3 or signalling occurs via FGFR1c. The BaF32 cells were used here to establish whether the different nanoparticles together with FGF2 in the absence of IL-3 enabled cell proliferation indicating the formation of ternary complexes capable of signalling. The formation of active ternary complexes between heparin, FGF2 and the FGFR1c present on the cell surface enabled BaF32 cell proliferation which was measured by the number of live cells by flow cytometry (Fig. 5A). Cells could only proliferate in the presence of both FGF2 and heparin (either Hep or L-Hep; positive controls) (Fig. 5A), while cells exposed to either FGF2 or heparin (negative controls) did not proliferate (Fig. 5A and Supplementary Fig. S4). Hep–nanoceria, L-hep–nanoceria-A and L-hep–nanoceria-B in the presence of FGF2 supported cell proliferation with a 3.5-, 3.8- and 3.1-fold increase in cell number (*P *<* *0.0001) compared to the negative controls (Fig. 5A). Furthermore, cells exposed to these same nanoceria preparations in the absence of FGF2 did not proliferate (Fig. 5A). The ability of the nanoparticles to form active ternary complexes with FGF2 and FGFR1c was due to the presence of heparin as nanoceria either in the presence or absence of FGF2 did not support BaF32 cell proliferation (Supplementary Fig. S4). These results also demonstrated that the heparin functionalization of nanoceria retained a similar level of biological activity as heparin alone as there was no significant difference (*P *>* *0.05) in cell number between these conditions (Figs. 5A and Supplementary Fig. S4) in line with previous studies using different heparin functionalized nanoceria preparations and analysis method [35, 48].

The higher heparin corona grafting density reduced BaF32 cell proliferation as shown by a 0.2-fold decrease in cell number in the presence of FGF2 and L-hep–nanoceria-B compared to FGF2 and L-hep–nanoceria-A (*P *=* *0.00002) (Fig. 5A). This result agrees with a previous study [48] which may be due to the reduced flexibility of heparin when grafted at a higher surface density. Furthermore, the number of BaF32 cells exposed to either hep–nanoceria or L-hep–nanoceria-A was not significantly different (*P *>* *0.05), indicating that the corona polymer length did not affect FGF2 signalling. This finding contrasts with the CAM assay results (Fig. 4A). However, the CAM represents a more complex model containing multiple endogenous heparin-binding growth factors and cell surface receptors. While it is possible that endogenous growth factors in the CAM contributed to the angiogenesis observed, the heparin functionalized nanoceria alone (200 µg/ml) did not promote angiogenesis suggesting that the dominant mechanism for angiogenesis was via the exogenous FGF2 and the heparin corona.

In a ternary FGF–FGFR–heparin complex, heparin binds FGF and FGFR via hydrogen bonds to enhance the affinity of FGF-FGFR complexes and promote dimerization for signalling [63]. The ternary complex activates signalling via the intracellular tyrosine kinase domain of FGFR through phosphorylation of specific tyrosine residues [62]. This signalling is terminated by the internalization of the FGF–FGFR complex followed by degradation in the lysosomes [61]. Thus, the fate of the nanoparticles after potentiating FGF2 signalling at the cell surface was explored (Fig. 5B and D). Flow cytometry indicated that there was no significant association of the nanoparticles with BaF32 cells after 24 or 72 h (Fig. 5B and C), which was confirmed by confocal microscopy (Fig. 5D). These findings suggest that nanoparticles interactions at the cell surface are transient and do not result in internalization of the nanoparticles and hence heparin internalization is not required for signalling via the receptor. These findings are consistent with the ability of the nanoparticles to promote angiogenesis in the CAM without internalization by endothelial cells (Fig. 3B). The high cell surface FGFR1c expression on the BaF32 cells may contribute to the inability of these cells to internalize the nanoparticles. The expression of FGFR1 on the cell surface projects a large part of this receptor into the extracellular space [64] and most likely restricted association of the nanoparticles that may interrupt endocytosis processes which are reported to dominate nanomaterial internalization [52, 65].

### The heparin corona reduces nanoceria trafficking to lysosomes in endothelial cells

In contrast to the BaF32 cells, which were exposed to nanoparticles concentrations up to 25 μg/ml without cytotoxicity (Fig. 5), nanoceria or heparin functionalized nanoceria doses > 1.5 μg/ml reduced primary endothelial cell viability (Supplementary Fig. S3). Thus, further *in vitro* experiments were performed with the nanoparticles at a dose of 1.5 μg/ml to analyse the effects in the absence of cytotoxicity.

**Figure 6.**
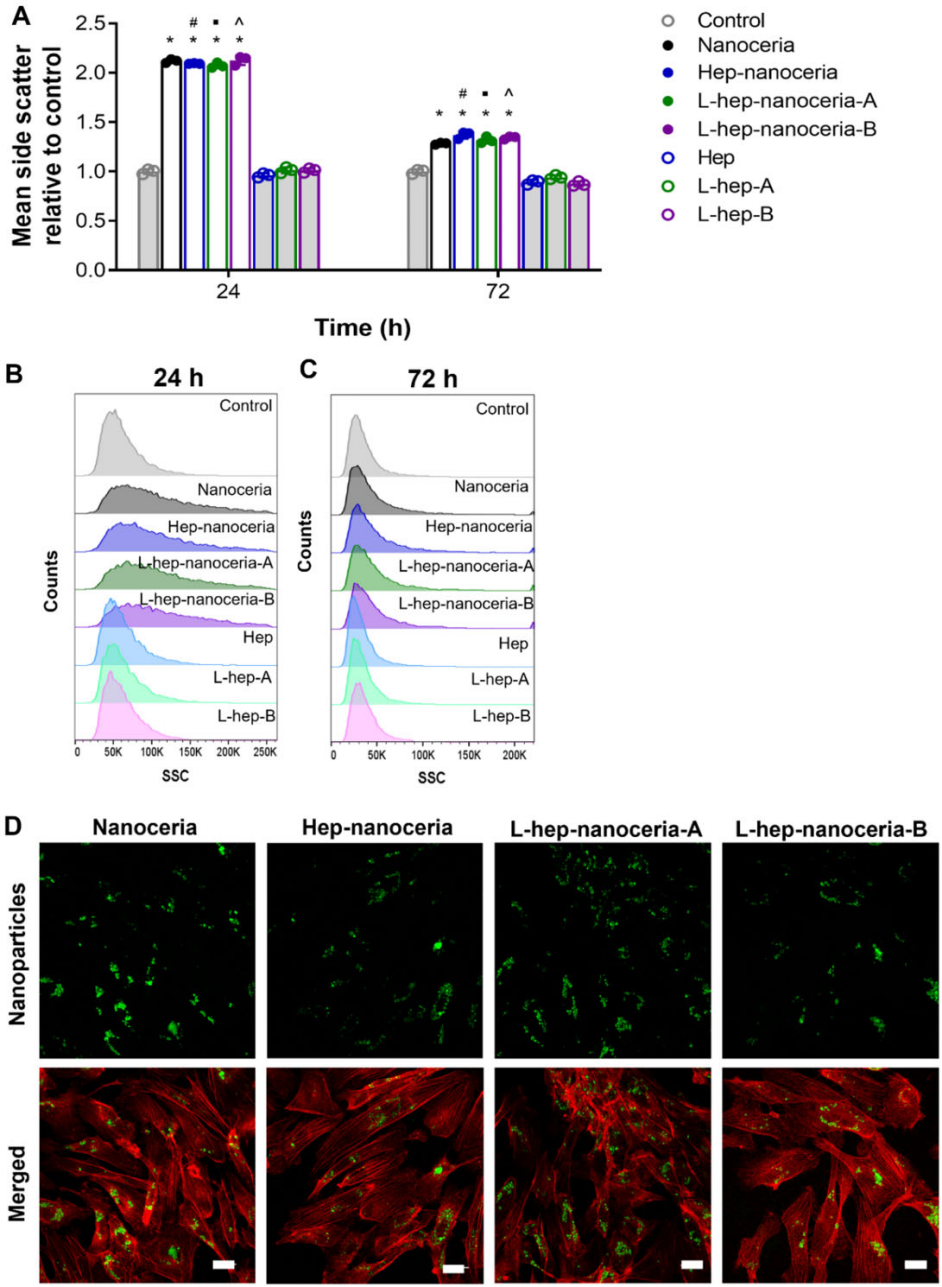
Nanoceria and heparin functionalized nanoceria associate with endothelial cells. (**A**) Flow cytometric analysis of the interaction of nanoceria, heparin functionalized nanoceria and heparin with HUVECs analysed by side scatter (SSC) after 24 and 72 h of exposure. Data were presented as fold change in SSC compared to control. Data are mean ± SD, *n* = 3, *P*‐values were calculated using two‐way ANOVA with Šidák’s test, *P <* *0.05 compared to control (medium), ^#^*P *<* *0.05 compared to hep, ^**•**^*P *<* *0.05 compared to L-hep-A and *^P *<* *0.05 compared to L-hep-B. Data was displayed as the number of cells versus SSC value histogram after (**B**) 24 h and (**C**) 72 h of exposure. (**D**) Representative images of the localization of nanoceria or heparin functionalized nanoceria (conjugated with AlexaFluor 488) within HUVECs stain for actin cytoskeleton after 24 h as measured by confocal microscopy. Scale bar = 10 μm.

Given the lack of association of the nanoparticles with the BaF32 cells after 24 h, it was of interest to explore the association with endothelial cells (Fig. 6A). The SSC measured by flow cytometry measures the granularity of the cells and was used to indicate nanoparticle internalization and/or binding to the cell surface [66]. The mean SSC for cells exposed to the nanoparticles was significantly higher (*P *<* *0.0001) compared to the control or heparin alone at both 24 and 72 h with a 2- and 1.3-fold increase, respectively (Fig. 6A). Confocal microscopy verified that endothelial cells internalized the nanomaterials (Fig. 6B). Interestingly, heparin functionalization did not affect the level of nanoceria association with the endothelial cells over 72 h (*P *>* *0.05) (Fig. 6A), which may be attributed to the low dose as heparin–nanoceria are reported to be internalized to a greater extent by activated human monocytes than nanoceria at doses of up to 200 μg/ml [66]. Similarly, the uptake of heparin-coated superparamagnetic iron oxide nanoparticles is reported to be concentration-dependent in human mesenchymal stem cells [67].

**Figure 7.**
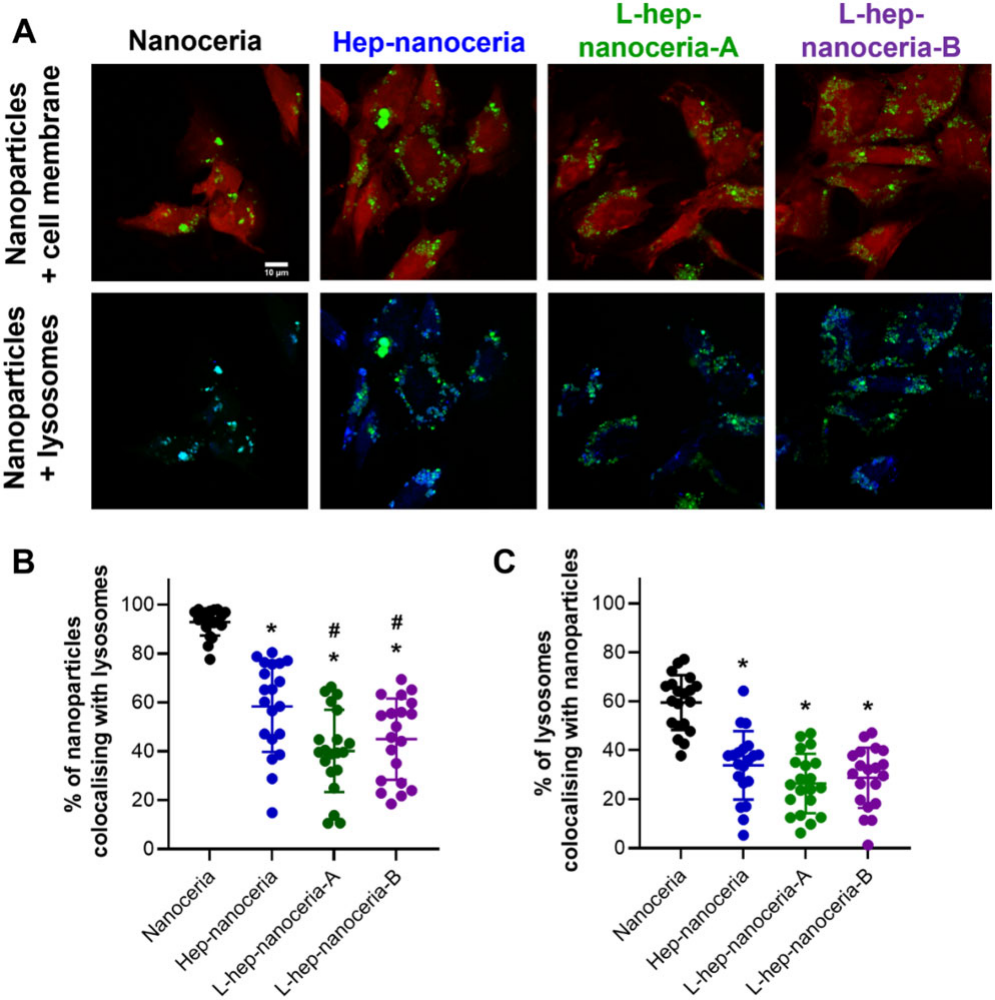
Colocalization of nanoceria or heparin–nanoceria with lysosomes in endothelial cells. (**A**) Colocalization of nanoparticles (green) merged with the cell membrane (red) and nanoparticle (green) merged with lysosomes (blue) in HUVECs imaged by confocal microscopy. Scale bar = 10 μm. Analysis of colocalization using the Manders’ correlation coefficient analysis with the proportion of (**B**) nanoparticles colocalizing with lysosomes and (**C**) lysosomes colocalizing with nanoparticles. Data are mean ± SD (*n* = 20), *p*-values were calculated using one-way ANOVA with Tukey’s test. * *P *<* *0.05 compared to nanoceria and ^#^*P *<* *0.05 compared to hep–nanoceria.

Further subcellular localization of the nanoparticles indicated colocalization with lysosomes (Fig. 7A) with ∼93% of internalized nanoparticles colocalized with lysosomes (Fig. 7B). Heparin functionalization of the nanoceria significantly (*P *<* *0.0001) reduced the extent of colocalization with lysosomes (Fig. 7B). This finding is supported by a previous study that reported cationic nanoceria colocalize with lysosomes to a greater extent than neutrally charged nanoceria [68]. Interestingly, the heparin chain length also affected the extent of colocalization with lysosomes with L-hep–nanoceria-A and L-hep–nanoceria-B exhibiting significantly (*P *=* *0.0017 and *P *=* *0.0345, respectively) less colocalization with lysosomes than Hep–nanoceria (Fig. 7B). Furthermore, the Manders’ colocalization analysis indicated that endothelial cells exposed to each of the nanoceria preparations contained lysosomes without nanoparticles. Interestingly, cells exposed to heparin functionalized nanoceria contained significantly less (*P *<* *0.0001) lysosomes with nanoparticles than cells exposed to nanoceria (Fig. 7C). These results indicated that heparin functionalization did not affect the extent of nanoparticle internalization; however, it affected the intracellular trafficking. Trafficking of nanoceria to lysosomes is associated with cytotoxicity [68]. Here, reduced lysosomal localization of the nanoceria was observed with the heparin surface corona; however, there was still an appreciate level of localization to lysosomes for these nanoparticles which may account for the cytotoxicity observed at doses >1.5 μg/ml. Furthermore, while anionic nanoceria are reported to exhibit reduced intracellular uptake compared to cationic or neutral nanoceria, their intracellular location was predominantly in the lysosome [68] as reported here.

Given the intracellular uptake of the nanoparticles, the ability to modulate intracellular ROS was of interest due to its role in angiogenic signalling pathways [19, 69]. However, when the nanoparticles were applied at 1.5 μg/ml, a dose which did not cytotoxic, there was no effect on the level of intracellular ROS *in vitro* (Supplementary Fig. S5A), which was further supported by an inability of the nanoparticles to affect the level of VEGFR2 expression (Supplementary Fig. S5B). This finding may be attributed to the low surface availability of Ce^3+^ and hence lower surface concentration of oxygen vacancies for ROS scavenging [26, 51] and further indicates that this dose of nanoceria did not promote angiogenesis via the ROS and VEGFR2-mediated signalling *in vitro* when in direct contact with cells [70, 71]. This finding contrasts with a previous report were heparin functionalized nanoceria reduced intracellular ROS in human coronary artery endothelial cells (HCAEC) [48]. The differences observed in the present study are likely due to the doses of nanoparticles as a dose of up to 50 μg/ml was not cytotoxic to HCAECs. Furthermore, the nanoparticles reported earlier were produced by flame spray pyrolysis and contained a lower level of heparin functionalization [48]. Moreover, there are likely cell type differences in toxicity to nanoceria as doses up to 200 and 400 μg/ml are cytocompatible for monocytes and melanoma cells, respectively [35, 36].

Together, this study demonstrates that a heparin surface corona on nanoceria promotes angiogenesis. While there was no effect of heparin chain length observed, the surface grafting density was positively correlated with angiogenic activity over the widest concentration range. Furthermore, the heparin surface corona on the nanoceria potentiated the activity of exogenous FGF2 *in vivo* and *in vitro*, with surface grafting density found to affect the extent of signalling. Internalization of the nanoparticles at cytocompatible doses by endothelial cells did not alter intracellular ROS levels, a property widely associated with nanoceria [15–18]; however, their localization in the lysosome, an acidic compartment, can be attributed to this finding and the extent of lysosomal trafficking was positively correlated with heparin chain length. These findings suggest that the angiogenic activity of heparin functionalized nanoceria are mediated in the extracellular environment *in vivo*; however, the *in vitro* assays favour the internalization of the nanoceria where they exhibit anti-angiogenic effects. These contrasting results indicate differences between the *in vitro* and *in vivo* models. Thus refined *in vitro* models, such as endothelial cells which display a glycocalyx on the cell surface as observed *in vivo*; an interface critically involved in nanoparticle transit across the cell membrane [72], will likely make inroads into further elucidating the mechanisms of the angiogenic activity of nanoceria with a heparin surface corona towards finding new treatments of tissue ischaemia.

## Supplementary data

Supplementary data are available at *REGBIO* online.

## Funding

We acknowledge funding support from the Australian Research Council (DP1097149) and UNSW Sydney. L.F. was supported by the University International Postgraduate Award (UIPA) at UNSW. Nanoparticle characterization including XPS, XRD and Raman were performed at the Mark Wainwright Analytical Centre, UNSW Sydney. TEM was performed at the Electron Microscope Unit (EMU). Confocal microscopy was performed at the Katharina Gaus Light Microscopy Facility, and flow cytometry and DLS were performed at Flow Cytometry Facility, which are also part of the Mark Wainwright Analytical Centre, UNSW Sydney, and in part-funded by the Research Infrastructure program at UNSW Sydney.

*Conflicts of interest statement*. None declared.

## Supplementary Material

rbac081_Supplementary_Data
