# Supplementary material for: Tuning the intentional corona of cerium oxide nanoparticles to promote angiogenesis via fibroblast growth factor 2 signalling
Source: Regen Biomater. 2022 Oct 20;9:rbac081. doi: 10.1093/rb/rbac081 (PMC9632454; doi:10.1093/rb/rbac081)
Supplement: rbac081_Supplementary_Data [file rbac081_supplementary_data.docx]

**Supplementary Information**

**Tuning the intentional corona of cerium oxide nanoparticles to promote angiogenesis via fibroblast growth factor 2 signalling**

Lu Fu^1^, Rupeng Li^2^, John M. Whitelock^1^, Megan S. Lord^1^*

| Sample | Weight loss (%) |
| --- | --- |
| Nanoceria | 3.9 |
| APTES-nanoceria | 9.0 |
| Hep-nanoceria | 11.8 |
| L-hep-nanoceria-A | 9.8 |
| L-hep-nanoceria-B | 12.6 |

**Supplementary Figure 1. Extent of nanoceria surface functionalisation with APTES and heparin determined by TGA.** The mass loss before 100 ℃ was regarded as dehydration. The weight loss of 3.93 % of nanoceria was due to the loss of surface hydroxyl groups. The loss in weight of the APTES-nanoceria was 5.10 % compared with the nanoceria, which equated to approximately 265 μmol/g particles. The weight losses of 2.7 %, 0.7 % and 3.6 % for Hep-nanoceria, L-hep-nanoceria-A and L-hep-nanoceria-B, respectively, compared to APTES-nanoceria were due to the loss of surface heparin groups.

**Supplementary Figure 2. Hydrodynamic size and surface distribution of nanoceria and heparin functionalised nanoceria.** (A) Size distribution (intensity) measured by DLS in PBS, pH 7.4 and (B) Zeta potential measured in MilliQ water, pH 7 of nanoceria (black), Hep-nanoceria (blue), L-hep-nanoceria-A (green) and L-hep-nanoceria-B (purple).

**Supplementary** **Figure 3. Dose-dependent cytotoxicity of nanoceria to endothelial cells.** **(A)** Endothelial cell (HUVEC) number measured after exposure to 0.1 – 200 µg/mL nanoceria or heparin functionalised nanoceria for 24 h compared to cells exposed to medium (control) measured by the CyQuant assay. 30 % cell growth inhibition level is indicated by the dashed line. **(B)** Endothelial cell (HUVEC) number measured after exposure to 1.5 µg/mL nanoceria or heparin functionalised nanoceria for 24 or 72 h compared to cells exposed to medium (control) measured by the CyQuant assay. Data are mean ± SD (n = 3), p‐values were calculated using one‐way ANOVA with Tukey's test and found to be p > 0.05 compared to control.

**Supplementary Figure 4. Activity of Hep, L-hep and nanoceria determined by the signalling of FGFR1c expressing BaF32 cells in the presence of FGF2.** Cells in the presence of FGF2 and heparin (either hep or L-hep; positive controls) were compared to cells in either the absence or presence of FGF and exposed to medium or nanoceria after 72 h. Nanoceria (H), nanoceria (A) and nanoceria (B) represent the same about of nanoceria as present in the conditions shown in Figure 7 A (Hep-nanoceria, L-hep-nanoceria-A and L-hep-nanoceria-B, respectively). Data presented as fold change in live cell number analysed by flow cytometry compared to cells seeded in each well at 0 h (mean ± SD, n= 3). *p*-values were calculated using a one-way ANOVA with Tukey’s test. **p* < 0.05 compared to cells with FGF2. ^#^*p* < 0.05 compared to cells exposed to each of the treatments in the absence of FGF2.

**Supplementary Figure 5. Nanoceria and heparin-nanoceria do not affect intracellular ROS level or VEGFR2 expression by endothelial cells.** **(A)** Analysis of intracellular ROS in endothelial cells (HUVEC) determined by flow cytometry after 24 h of exposure to the nanoparticles or heparin compared to cells exposed to medium. The level of intracellular ROS was measured using the intracellular ROS-dependent oxidation of DCFH-DA. **(B)** Analysis of VEGFR2 expression by HUVECs determined by flow cytometry after 24 h of exposure to the nanoparticles or heparin compared to cells exposed to medium. PI was used to gate for live cells. Data was analysed using FlowJo v10 software and displayed as the number of cells versus fluorescence intensity.

**Supplementary Table 1. Proportion of cerium ions, Ce^3+^ or Ce^4^in nanoceria and heparin functionalised nanoceria.**

| **Sample** | **Proportion (%)** | | **Ce^3+^/Ce^4+^ ratio** |
| --- | --- | --- | --- |
|  | **Ce^3+^** | **Ce^4+^** |  |
| Nanoceria | 35.2 | 64.8 | 0.54 |
| Hep-nanoceria | 35.7 | 64.3 | 0.55 |
| L-hep-nanoceria-A | 36.9 | 63.2 | 0.58 |
| L-hep-nanoceria-B | 37.0 | 63.0 | 0.59 |

**Supplementary Table 2. Composition of nanoceria and heparin functionalised nanoceria determined by XPS.**

| **Element** | **Atomic %** | | | | | **Mass %** | | | | |
| --- | --- | --- | --- | --- | --- | --- | --- | --- | --- | --- |
|  | **Nanoceria** | **APTES-nanoceria** | **Hep-nanoceria** | **L-hep-nanoceria-A** | **L-hep-nanoceria-B** | **Nanoceria** | **APTES-nanoceria** | **Hep-nanoceria** | **L-hep-nanoceria-A** | **L-hep-nanoceria-B** |
| **Ce** | 19.9 | 9.12 | 12.3 | 11.7 | 8.5 | 69.9 | 48.3 | 56.4 | 54.9 | 46.5 |
| **O** | 68.3 | 48.5 | 57.1 | 55.5 | 50.4 | 27.4 | 29.2 | 29.8 | 29.8 | 31.4 |
| **C** | 8.8 | 28.2 | 20.0 | 22.0 | 27.6 | 2.6 | 12.7 | 7.8 | 8.9 | 12.9 |
| **Si** | - | 5.8 | 3.4 | 3.2 | 3.8 | - | 6.1 | 3.1 | 3.0 | 4.2 |
| **S** | - | - | 1.1 | 1.3 | 1.8 | - | - | 1.1 | 1.4 | 2.3 |
| **N** | - | 6.9 | 3.8 | 4.1 | 4.9 | - | 3.7 | 1.8 | 1.9 | 2.7 |
